# Lytic and temperate phage naturally coexist in a dynamic population model

**DOI:** 10.1093/ismejo/wrae093

**Published:** 2024-05-31

**Authors:** Ofer Kimchi, Yigal Meir, Ned S Wingreen

**Affiliations:** Lewis-Sigler Institute for Integrative Genomics, Princeton University, Princeton, NJ 08544, USA; Department of Physics, Ben-Gurion University, Be’er Sheva 84105, Israel; Department of Physics, Princeton University, Princeton, NJ 08544, USA; Lewis-Sigler Institute for Integrative Genomics, Princeton University, Princeton, NJ 08544, USA; Department of Molecular Biology, Princeton University, Princeton, NJ 08544, USA

**Keywords:** phage, lysogen, ecology, model, virulent, lytic, temperate, coexistence

## Abstract

When phage infect their bacterial hosts, they may either lyse the cell and generate a burst of new phage, or lysogenize the bacterium, incorporating the phage genome into it. Phage lysis/lysogeny strategies are assumed to be highly optimized, with the optimal tradeoff depending on environmental conditions. However, in nature, phage of radically different lysis/lysogeny strategies coexist in the same environment, preying on the same bacteria. How can phage preying on the same bacteria coexist if one is more optimal than the other? Here, we address this conundrum within a modeling framework, simulating the population dynamics of communities of phage and their lysogens. We find that coexistence between phage of different lysis/lysogeny strategies is a natural outcome of chaotic population dynamics that arise within sufficiently diverse communities, which ensure no phage is able to absolutely dominate its competitors. Our results further suggest a bet-hedging mechanism at the level of the phage pan-genome, wherein obligate lytic (virulent) strains typically outcompete temperate strains, but also more readily fluctuate to extinction within a local community.

Phage—viruses that infect bacteria—are subject to strong evolutionary pressures. One optimization axis is the lysis–lysogeny decision phage face when infecting a bacterium. Upon infection, phage can either lyse the bacterium, generating a burst of phage progeny, or lysogenize it, incorporating the phage genome into the bacterium. The resulting lysogen is generally immune to subsequent infection by the same class of phage [1, 2].

Obligate lytic phage, such as *Escherichia coli* phage T4, never lysogenize their bacterial hosts; temperate phage, such as the *E. coli* phages λ and P1 and the *Vibrio cholerae* phage CTXφ, can perform either lysis or lysogeny upon infection [3]. The optimal lysis/lysogeny tradeoff depends on environmental conditions [4, 5]: given abundant susceptible bacteria, phage do better by performing lysis; when these bacteria are scarce, phage are better off lysogenizing their hosts [6]. However, lytic and temperate phage that prey on the same bacteria coexist with one another [7, 8]. How can they coexist if one is more optimal than the other?

Explanations for the coexistence of competing species generally rely on the idea that different species have different ecological niches, e.g. in terms of resources or space [9, 10]. In contrast, here we show that naturally arising chaotic population dynamics are sufficient for the coexistence of obligate lytic and temperate phage. These dynamics arise only within sufficiently diverse communities and are absent when a single obligate lytic and temperate strain compete.

We consider $N_c$ phage classes preying on a single bacterial species (Fig. 1). Lysogens are immune to reinfection by a phage of the same class. Each phage population density $P_{cf}$ is indexed by its immunity class $c$ and strain $f$, i.e. the fraction of its infections leading to lysogeny. Strains with $f=0$ are obligate lytic and have no associated lysogens. The population densities of phage $P_{cf}$ and their associated lysogens $L_{cf}$ change according to (see Fig. 1 for parameter definitions and more detail):


(1) \begin{equation*} \frac{d{P}_{cf}}{dt}=\underset{\mathrm{Induction}}{\underbrace{b\gamma{L}_{cf}}}-\underset{\mathrm{Phage}\ \mathrm{death}\ \mathrm{and}\ \mathrm{adsorption}}{\underbrace{\left(\delta +k\ {\sum}_{c^{\prime }}{L}_{c^{\prime }}\right){P}_{cf}}}+\underset{\mathrm{Phage}\ \mathrm{growth}\ \mathrm{through}\ \mathrm{lysis}}{\underbrace{kb\left(1-f\right){P}_{cf}{\sum}_{c^{\prime}\ne c}{L}_{c^{\prime }}}}, \end{equation*}

$$\frac{d{L}_{cf}}{dt}=\underset{\mathrm{Lysogen}\ \mathrm{growth}}{\underbrace{\alpha{L}_{cf}}}-\underset{\mathrm{Induction}}{\underbrace{\gamma{L}_{cf}}}-\underset{\mathrm{Lysogen}\ \mathrm{death}\ \mathrm{due}\ \mathrm{to}\ \mathrm{lysis}}{\underbrace{k{L}_{cf}{\sum}_{c^{\prime}\ne c}{\sum}_{f^{\prime }}\left(1-{f}^{\prime}\right){P}_{c^{\prime }{f}^{\prime }}}} ,$$

**Figure 1:**
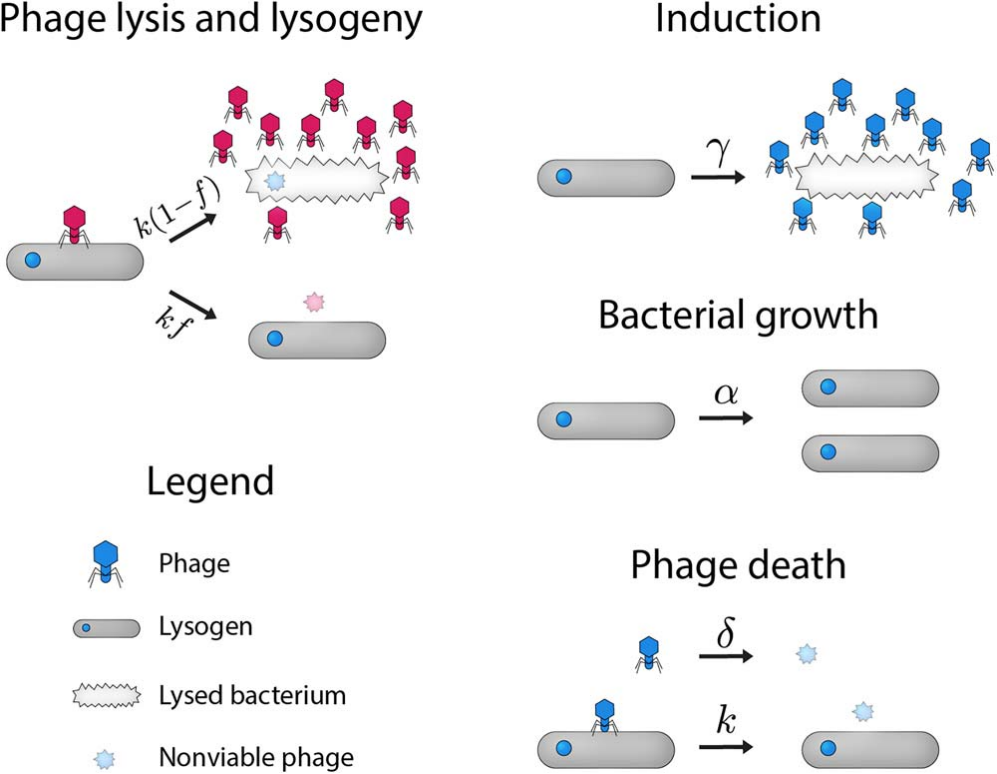
Model overview. A pictorial representation of the simplified model described by equations (1). Phage of different immunity classes are represented by different colors. Here, we disallow double lysogens and treat the population of sensitive (non-lysogenic) bacteria as negligible (see main text and Supplementary Section S2). Bacteria grow at a rate $\alpha$, and are limited by phage predation rather than resource limitation. Phage populations grow through lysis. Phage infect bacteria at a rate $k$, leading to either lysis or lysogeny. Phage strains within a class are distinguished by their (fixed) fraction of infections that lead to lysogeny, denoted by $f$. If the phage performs lysis, it creates $b$ new copies of the phage and kills the bacterium. Lysogens are immune to reinfection by a phage of the same class, and are spontaneously induced to undergo lysis at a rate $\gamma$. Phage die (or migrate away) at a rate $\delta$, and phage that attempt to infect immune lysogens also die. Values for parameters are motivated by [11, 12] (see Supplementary Section S1).

where ${L}_c={\sum}_f{L}_{cf}$. Equations (1) are a natural simplification of more comprehensive models which produce similar results; see Supplementary Sections S2 and S3, and Fig. S1. Although we focus on symmetric systems for simplicity and clarity, our results throughout are robust to heterogeneities in all parameters (Figs S3 and S12).

When a single obligate lytic strain and a single temperate strain compete, one or the other goes extinct depending on whether they are of the same or different classes (Fig. S2). However, when multiple temperate strains compete, we observe sustained chaotic dynamics (Figs 2a and S3a; Supplementary Section S4). These chaotic dynamics are robust to variations in parameter values and parameter heterogeneity, with almost no simulations resulting in steady-state behavior (Figs S10, S11, S12). We hypothesized that these frequent large variations in populations (or “boom and bust” cycles) could lead to an opportunity for obligate lytic phage.

**Figure 2:**
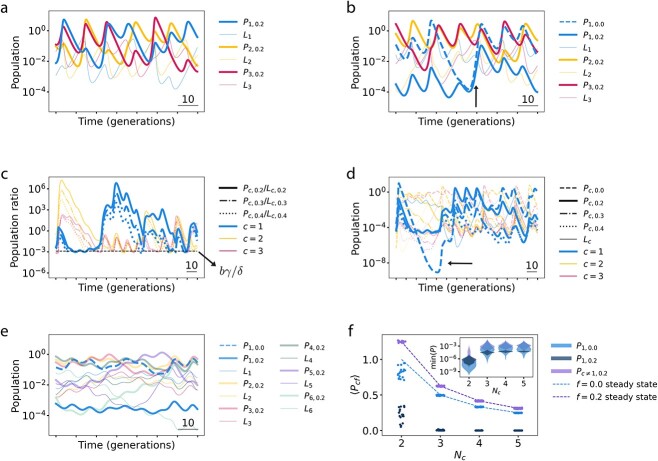
Population dynamics of coexisting obligate lytic and temperate phage. (**A**) Competition among multiple temperate phage classes ($N_c=3$) leads to chaotic dynamics. (**B**) Chaos allows obligate lytic and temperate phage to coexist, with obligate lytic strains typically dominating over temperate strains of the same class. Arrow points to a growth period of phage of immunity class 1 (blue). (**C**) Simulation of $N_c=3$ phage immunity classes each with one obligate lytic strain and three temperate strains; all strains coexist together. Here, temperate phage population densities are plotted normalized by their respective lysogen population density. In periods of growth of a particular phage immunity class, the more lytic strains dominate, but phage-to-lysogen population density ratios “bunch” together at troughs, with a population density floor predicted by equation (2) (black dashed line). (**D**) Plot of population densities (rather than population density ratios) of simulation shown in (**C**). In order to compare the temperate phage population density floor with a population dip of an obligate lytic phage which lacks such a floor (arrow), lysogens of different strains of the same class were initialized with equal population densities (and remain equal indefinitely; see Fig. S4). (**E**) Increasing the number of phage classes (here to $N_c=6$) generally leads to smaller population density variation. (**F**) Summary statistics across 50 simulations, each for 2000 generations. Each simulation competes ${N}_c-1$ immunity classes, each consisting of a single temperate strain (purple), along with a single class with two strains: One obligate lytic (light blue), one temperate (dark blue). The steady-state fixed-point solutions for the phage population densities (equation (3); dashed curves) agree well with the average of the chaotic trajectories. Average phage population densities decrease with $N_c$ (main figure, scatter plot) whereas minimum population densities increase with $N_c$ (inset, violin plot). Simulations for $N_c=2$ in which the obligate lytic phage fluctuated to extinction were excluded.

Obligate lytic strains are best able to capitalize on periods of phage population growth, outcompeting temperate strains of the same immunity class because they turn all bacterial hosts they infect into new phage (Fig. 2b; arrow). Indeed, when an obligate lytic strain was introduced into the simulations of Fig. 2a, it typically dominated over the temperate strain of the same class (Fig. 2b). At the same time, both strains persisted at high population densities, with little change in the behavior of phage of other immunity classes.

How do temperate phage persist if obligate lytic phage outcompete them during periods of phage expansion? We carried out simulations with three phage immunity classes, each with four strains (i.e. different values of $f$). We continued to see robust coexistence among all strains, and noticed a striking “bunching” effect when plotting the ratio of the temperate phage to their corresponding lysogen population densities (Fig. 2c). During periods of growth of a particular phage class, strains with smaller lysogeny fractions $ f$ typically outcompete those with larger $f$; however, $P/L$ ratios were all nearly equal at the troughs (Fig. 2c). We traced this bunching effect to the induction of lysogens, which buffers temperate phage populations against periods of decline. To test this explanation quantitatively, we consider the behavior of an obligate lysogenic (or “dormant”) phage with $f=1$, whose dynamics are entirely determined by its respective lysogen. By setting $dP_{c1}/dt=0$, we find a homeostatic population density for the phage, ${P}_{c1}^{\ast }$, which is approximately proportional to the strain’s lysogen population density:


(2) \begin{equation*} \frac{P_{c1}^{\ast }}{L_{c1}}=\frac{b\gamma}{k\ {L}_c+\delta }. \end{equation*}

When the phage population density dips below ${P}_{c1}^{\ast }$, induction restores it back up, and when the phage population density rises above ${P}_{c1}^{\ast }$, phage death pushes it down. Equation (2) predicts the population densities of temperate phage at the troughs with reasonable accuracy (Fig. 2c); in fact, an even further simplified estimate of $b\gamma /\delta$ only overestimates the minimum population density ratios by a factor of ~2, compared to the overall variation in $P/L$ of $\mathcal{O}\left({10}^9\right)$ (We note that the simplified model of equations (1) retains a perfect memory of the initial ratios of lysogens of the same immunity class because $dL_{cf}/ dt$ is independent of $f$. This feature is incidental to the observed bunching effect; see Fig. S4.) The bunching effect, therefore, implies a population density “floor” for the temperate phage, protecting their populations during periods of decline.

In contrast, there is no population density floor for the obligate lytic phage, which have no corresponding lysogens, and therefore no induction buffering their populations (Fig. 2d). Therefore, although obligate lytic strains typically outcompete temperate strains, lytic strain population densities occasionally drop to very low levels. What then protects obligate lytic strains in nature from going extinct over long times?

In our model, the presence of more competitors leads to more stable behavior for all phage, raising the minima of obligate lytic strain populations. This effect arises because population minima are tied to the magnitude of fluctuations in the sum of the resources available to each phage; these fluctuations decrease as the number of distinct lysogenic species on which each phage can prey is increased (Figs 2e and S5). To explore this effect, we consider ${N}_c-1$ classes each consisting of a single temperate strain, along with a single class with two strains (one obligate lytic and other temperate). Although the system dynamics are chaotic, the average population density $\left\langle{P}_{cf}\right\rangle$ of each phage with one strain in its immunity class agrees quantitatively with the steady-state fixed-point value (see Supplementary Section S5),


(3) \begin{equation*} {P}_{cf}^{\mathrm{ss}}=\frac{\alpha -\gamma }{k\ \left({N}_c-1\right)\left(1-f\right)}, \end{equation*}

i.e. the non-zero solution to $dP/ dt= dL/ dt=0$ (Fig. 2f). The average of the obligate lytic phage population density is also reasonably well predicted by equation (3) with $f=0$, despite the presence of a temperate strain of the same immunity class (Fig. 2f). The obligate lytic population density is on average orders of magnitude larger than that of the temperate phage in its immunity class, but only slightly smaller than the population densities of temperate phage of other immunity classes, as predicted by equation (3). Also as predicted by equation (3), as $N_c$ is increased, the average population density of each strain decreases; however, the minimum population density of each strain *increases* and then plateaus with increasing $N_c$ (Fig. 2f, inset). Thus, extinction becomes less likely in communities consisting of more phage immunity classes.

Our model makes several experimentally testable predictions. These can be tested with a community of several phage immunity classes, created following e.g. reference [2]. First, we predict chaotic population fluctuations when a sufficient number of phage of different immunity classes are placed within a chemostat with dilution rate slower than the phage-induced death rate of lysogens. Second, introducing an obligate lytic phage in the same immunity class as one of the temperate phages will lead to a substantial (albeit finite) decrease of the latter’s population, with little effect on the behavior of the phage in other immunity classes. Third, obligate lytic phage will reach lower population minima than temperate phage. Finally, we predict that whereas the average phage populations will decrease with increased number of immunity classes $N_c$, the minimum phage populations taken over a long enough window will actually increase with $N_c$.

To address the robustness of these predictions to our simplifying approximations, we construct extensions of our model to more realistic systems including sensitive bacteria and double lysogens (Figs S6 and S7), heterogeneous lysogeny probabilities (Fig. S3b-c), obligate lytic phage with no temperate strain of the same immunity class (Fig. S3d), and a finite lysis time and coinfection-induced lysogeny [13, 14] (Fig. S8), all of which yield the same qualitative behavior. Finally, although we have focused on ecological dynamics in the absence of mutations, we expect our results to hold in laboratory experiments. Whereas bacterial populations can in some cases rapidly develop phage-resistant mutations, these are typically followed by compensatory mutations in the phage [15, 16]. Furthermore, resistance mutations typically carry a cost to bacteria such that they do not always arise in natural populations containing multiple phage strains [17]. In particular, the presence of other competing bacteria could thus restrict the survival of bacteria with costly phage-resistance mutations. Similarly, although phage can over time evolve to infect new hosts, we expect these evolutionary dynamics to be much slower than the ecological dynamics considered here [18]. Nevertheless, further studies modeling simultaneous time-dependent ecological dynamics and evolution may fruitfully reveal interactions between these dynamics.

Contrary to prior work finding, optimal phage strategies survive as sub-optimal strategies go extinct [11, 19], our results suggest coexistence of different strategies may be commonplace. With a single obligate lytic phage strain and a single temperate phage strain of different immunity classes (i.e. a model akin to that considered in Fig. S2, bottom row), prior work showed static steady-state coexistence is possible for certain parameter combinations, such as when the obligate lytic phage burst size and infection rate are smaller than those of the temperate phage [20]; steady-state coexistence was also seen for phage of the same immunity class [21]. Our work expands beyond these scenarios, finding for multiple classes a qualitatively different form of coexistence mediated by chaos. Although in principle, a steady-state fixed point can be found as a solution to equation (1) (see equation (3)), its basin of attraction is so small that we predict any real instantiation of such a system will undergo robust chaotic dynamics (see also Supplementary Section S4 and Figs S10 and S11). Species coexistence mediated by chaotic population dynamics has been studied theoretically in other contexts including generalized Lotka-Volterra models and has been shown to arise from heterogeneities of large systems [10, 22, 23]; here, chaos arises naturally from the interactions of phage with their lysogens and is robust to varying degrees of parameter heterogeneity, including a lack thereof (Fig. S12).

Our results suggest a natural bet-hedging mechanism for phage on the pan-genome level. When susceptible bacteria are plentiful, obligate lytic strains thrive, while their temperate cousins of the same immunity class persist at lower populations. When conditions worsen, however, temperate strains can outcompete obligate lytic strains. Thus, obligate lytic strains, typically dominant, would be first to go extinct. Here, the bet-hedging is not a product of the behavior of individual organisms, but rather a feature of competing strains within a larger, genetically related, population.

## Supplementary Material

KimchiMeirWingreen_Supp_Final_wrae093

## Data Availability

Data sharing not applicable to this article as no datasets were generated or analysed during the current study.

## Code availability

All code used to generate the results and figures in this study can be found at https://github.com/ofer-kimchi/lytic-temperate-coexistence.
