# Supplementary material for: Lytic and temperate phage naturally coexist in a dynamic population model
Source: ISME J. 2024 May 31;18(1):wrae093. doi: 10.1093/ismejo/wrae093 (PMC11187991; doi:10.1093/ismejo/wrae093)
Supplement: KimchiMeirWingreen_Supp_Final_wrae093 [file kimchimeirwingreen_supp_final_wrae093.pdf]

Supplementary Information

Supplemental figures

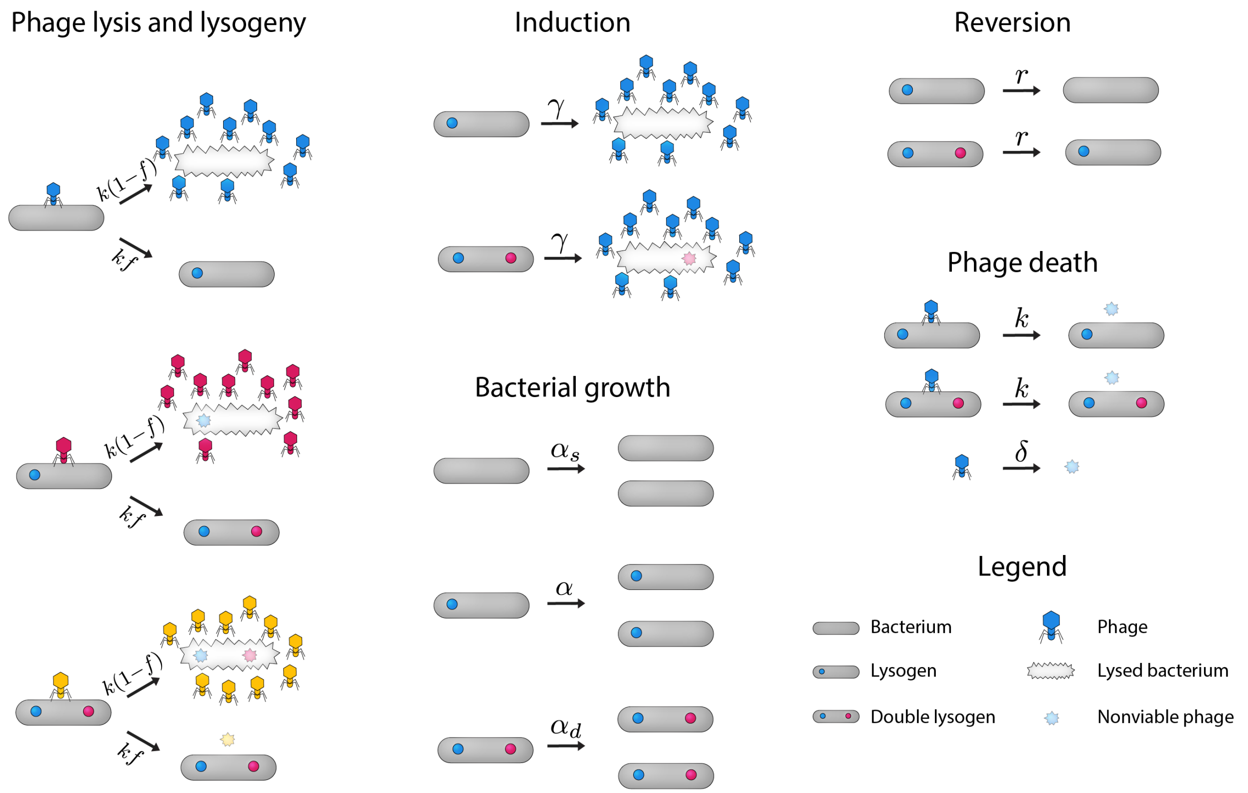

Figure S1: **Comprehensive model overview.** A pictoral representation of the full model described by equations (S3). Phage of different immunity classes are represented by different colors.

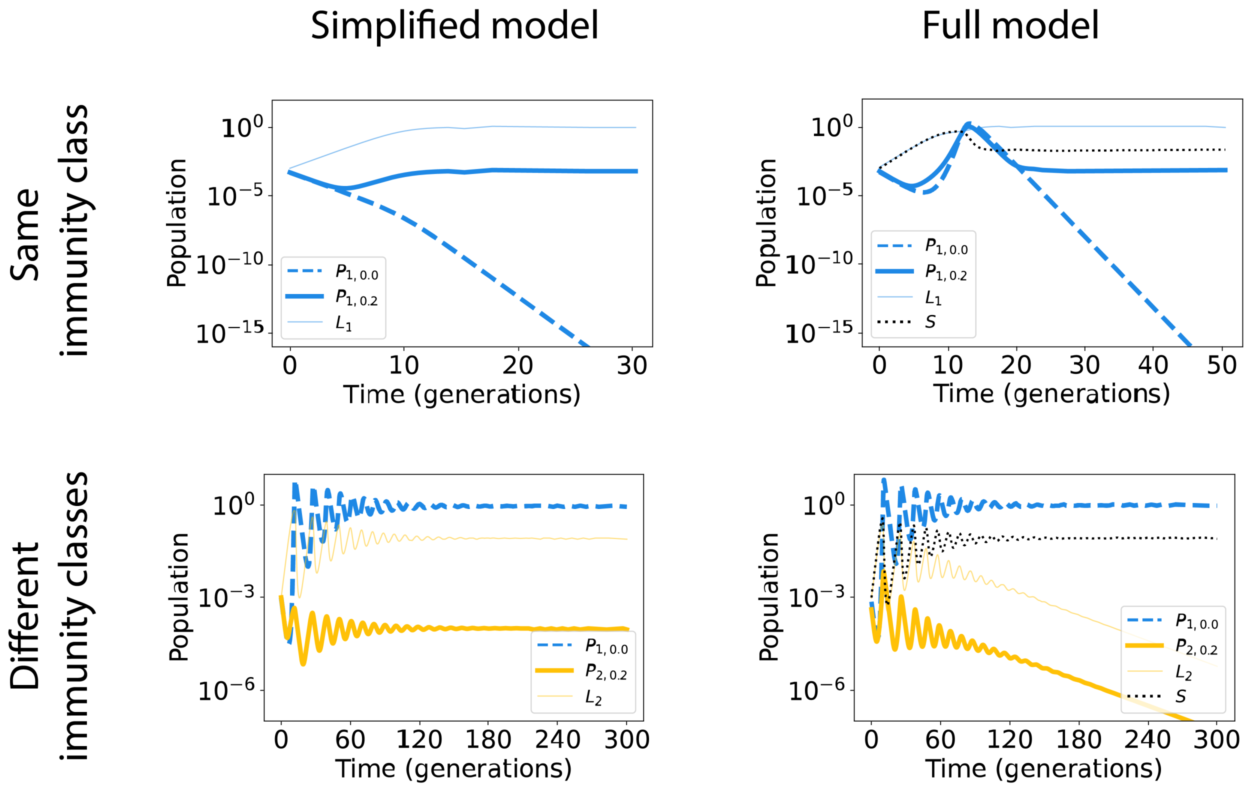

Figure S2: **Competition between a single obligate lytic strain and a single temperate strain.** We show the results of competition between a single obligate lytic strain and a single temperate strain of either the same (top row) or different (bottom row) immunity classes. Both the simplified (left column; equations (1)) and more comprehensive (i.e. including sensitive bacteria; Eqs. (S3) and (S4); right column) models show that when the two strains are of the same immunity class, the obligate lytic strain goes extinct, while the temperate strain survives. When the two strains are of different immunity classes, the obligate lytic phage dominates over the temperate phage as the temperate-phage lysogens are not immune to the lytic phage. In the comprehensive model, the sensitive strain outcompetes the lysogens as the former has a slightly higher growth rate, leading the temperate strain in that model to go extinct; in the simplified model, lysogens survive and so the temperate strain persists as a result of induction. In equations (S3) and (S4) for the full model, the carrying capacity of bacteria was set to $K = 1$.

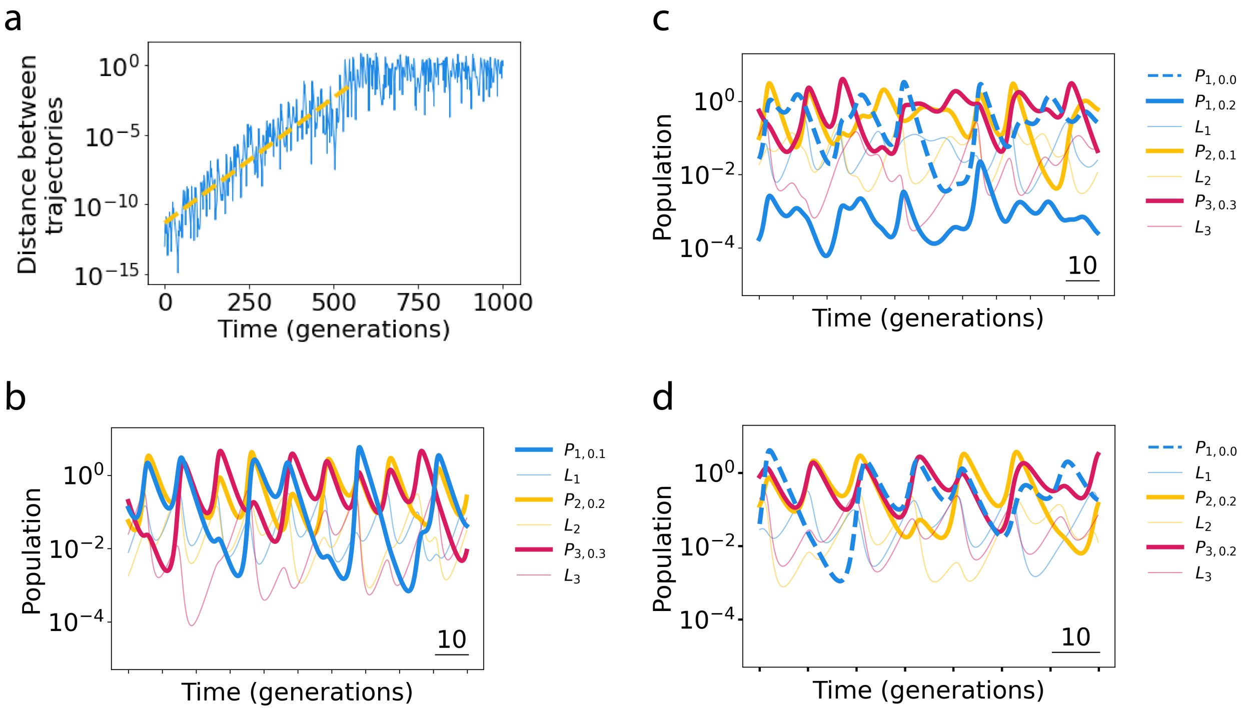

Figure S3: **Trajectories display robust chaotic dynamics. a,** Three temperate phage of different immunity classes were simulated. Then, a second simulation with nearly identical initial conditions was run (initial phage population densities were set to be 10^{-13} larger). We plot in blue the distance between the two trajectories of the first immunity class, $\sqrt{(P_{1,0.2} - P'_{1,0.2})^2}$. The initial portion of the plot is fit to an exponential, with Lyapunov exponent 6×10^{-2} (yellow dashed line). That nearby trajectories diverge exponentially is a hallmark of chaotic dynamics. **b, c,** Chaotic dynamics persist with heterogeneous lysogeny fractions f, with little qualitative difference from the case of homogeneous f (Fig. 2a-b). **d,** Chaotic dynamics are qualitatively unchanged if the obligate lytic strain is not paired with a temperate strain of the same immunity class. Here, bacteria labeled L_1 are non-lysogenic (with induction rate $\gamma = 0$) but are immune to infection by phage $P_{1,0}$.

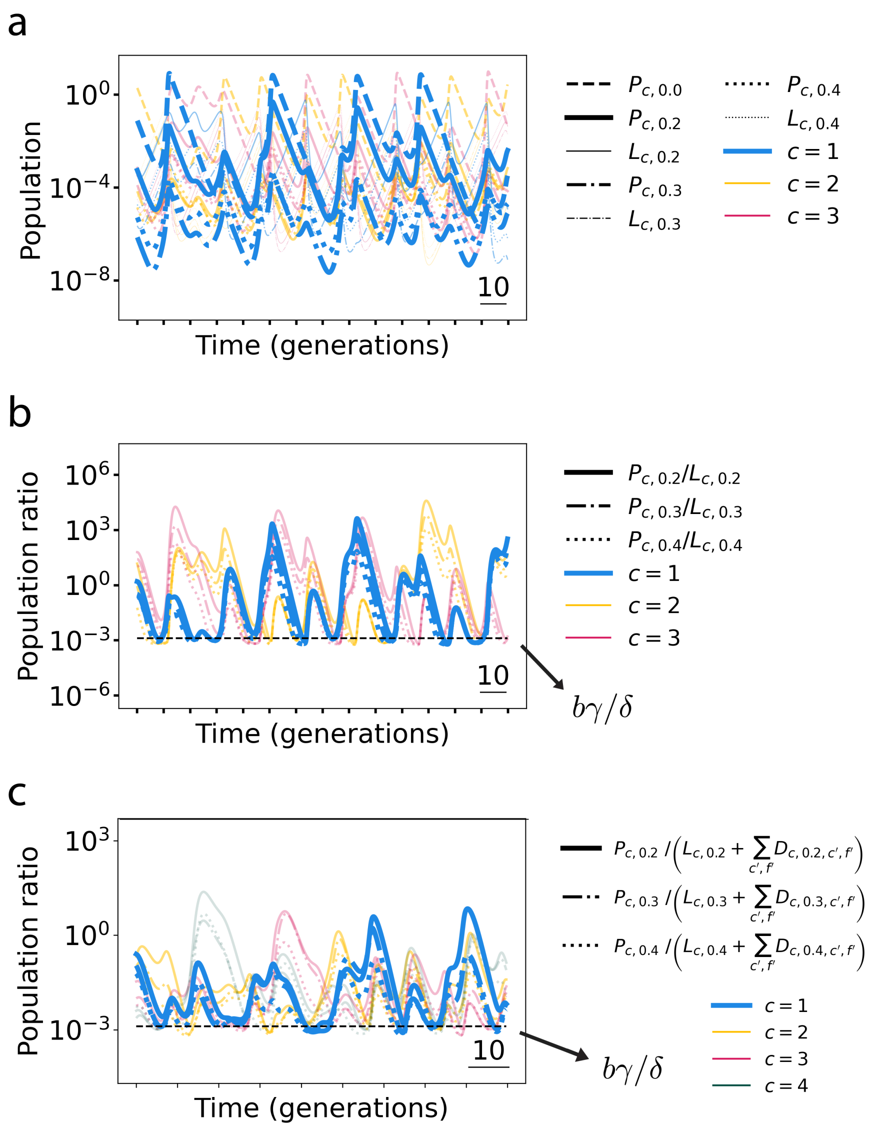

Figure S4: **Bunching effect with heterogeneous initial lysogen population densities. a,** Simulations performed as in Fig. 2c-d, i.e. with $N_c = 3$ phage immunity classes each with one obligate lytic strain and three temperate strains, but with one difference: here, initial lysogen population densities were chosen randomly over a range of ~ 2 orders of magnitude. Since in the simplified model, equations (1), $dL_{c,f}/dt$ is independent of f, the lysogens of different strains of the same immunity class vary in lockstep together, and the system retains a perfect memory of their initial population density ratios. **b,** The bunching effect at phage population troughs is independent of this memory, and the ratio of the population density of each phage to the population density of its respective lysogen is qualitatively unchanged compared to Fig. 2c. **c,** The full model (equations (S3)), which lacks this memory, displays the same bunching effect; furthermore, the population density floor is quantitatively unchanged from the simplified model, and is determined by the total population density of each phage strain's respective lysogens, including both single and double lysogens.

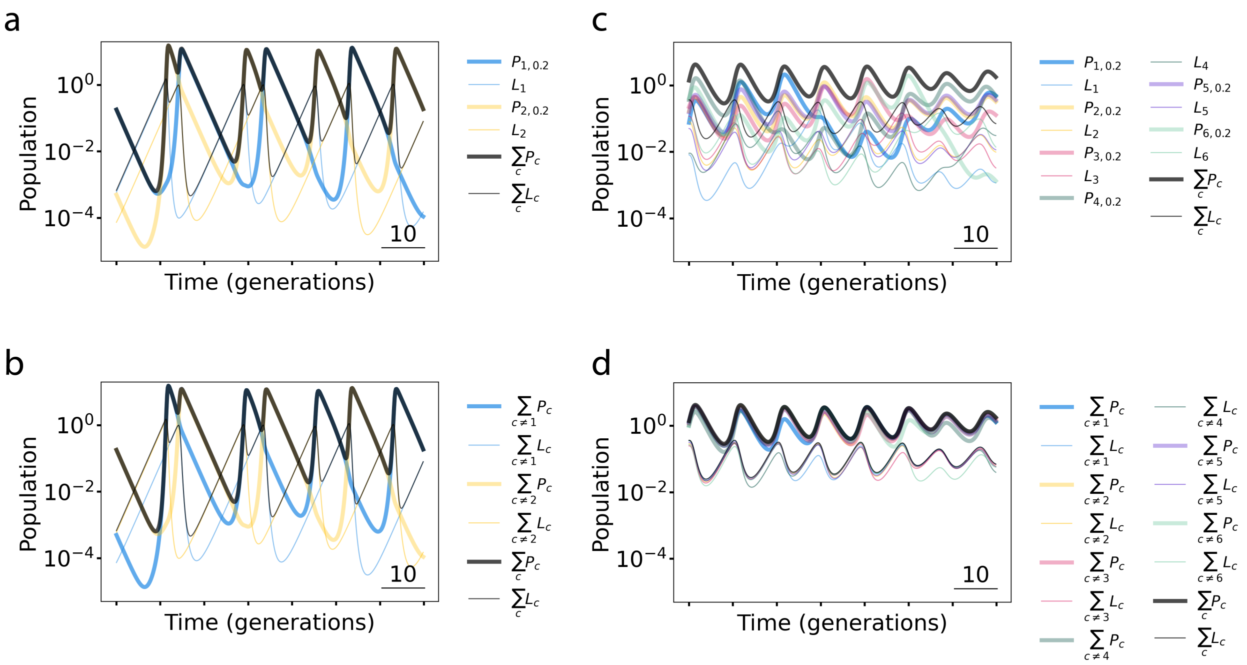

Figure S5: **Fluctuations decrease with more competing phage strains. a,c,** Representative simulations with two (panel a) or six (panel c) phage immunity classes showing fluctuations in both individual (colors) and overall (black) population densities. **b,d,** The same simulations as in panels a and c but with the summed population densities of all immunity classes but one visualized as distinct colors, demonstrating a decrease in variability with six immunity classes (panel d) compared to two (panel b).

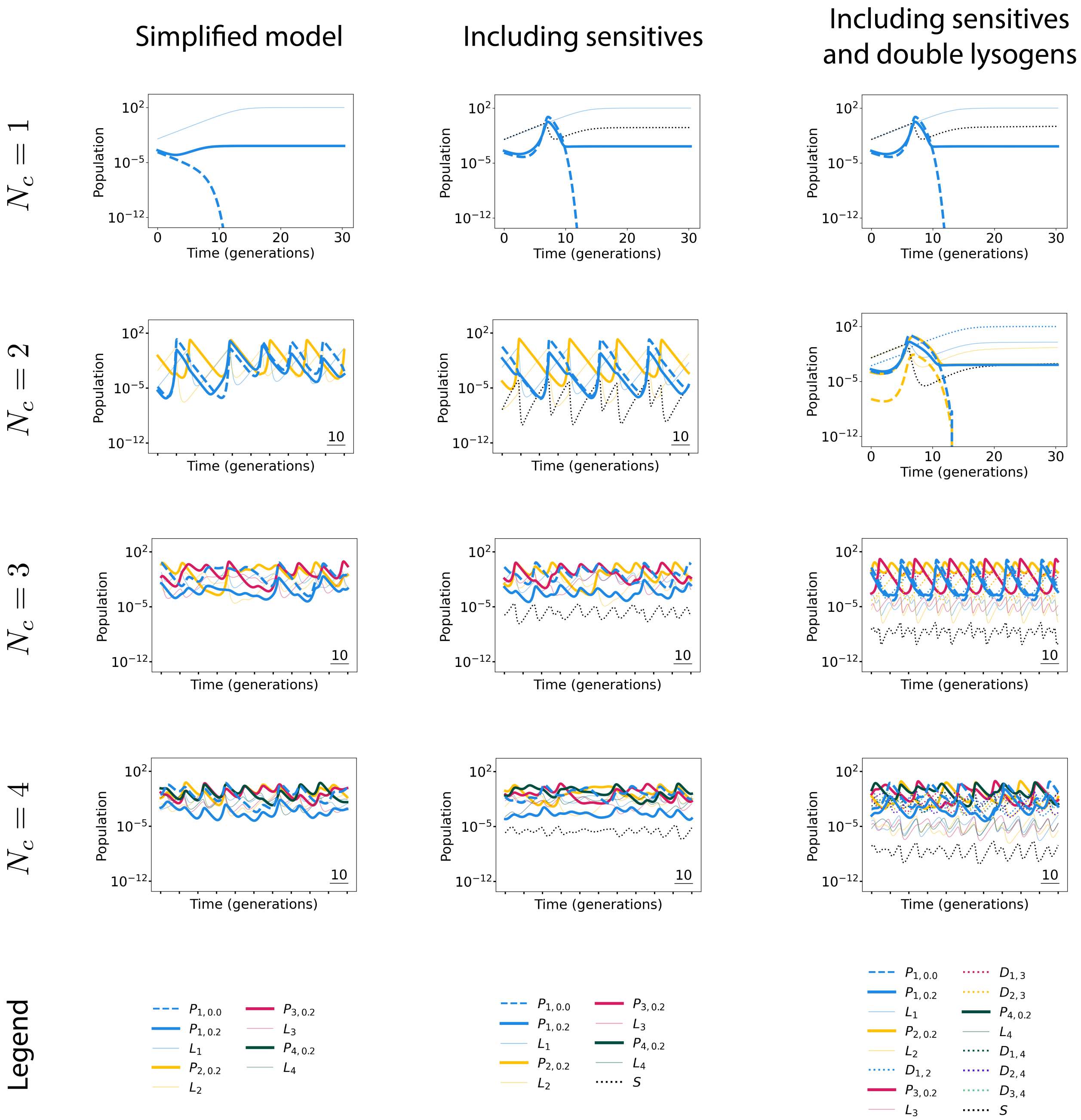

Figure S6: **Comparison of models with different degrees of simplification.** Simulation results are shown for the simplified model (equations (1), left column), the model including sensitive (i.e. non-lysogenic) bacteria (equations (S4), middle column), and the full model including both sensitive bacteria and double lysogens (equations (S3), right column). Each simulation has N_c competing phage immunity classes. One immunity class has both an obligate lytic strain and a temperate strain, while the other $N_c - 1$ immunity classes have a single temperate strain. Carrying capacity of bacteria, K, was set to 100 in the middle and right columns.

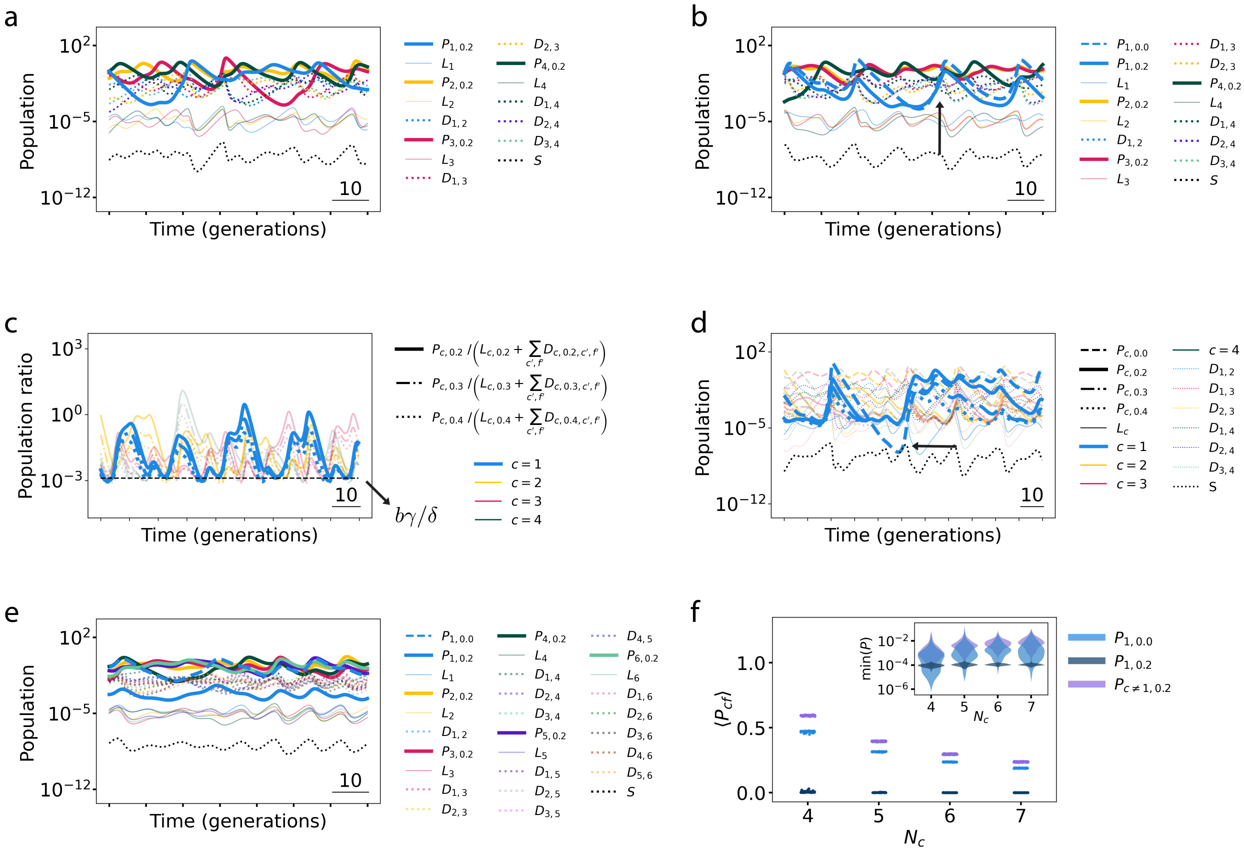

Figure S7: **Recapitulation of Fig. 2 using a more comprehensive model.** All panels as in Fig. 2, using equations (S3) in place of equations (1). $N_c = 2, 3$ are not included in panel f as they lead to oscillatory solutions.

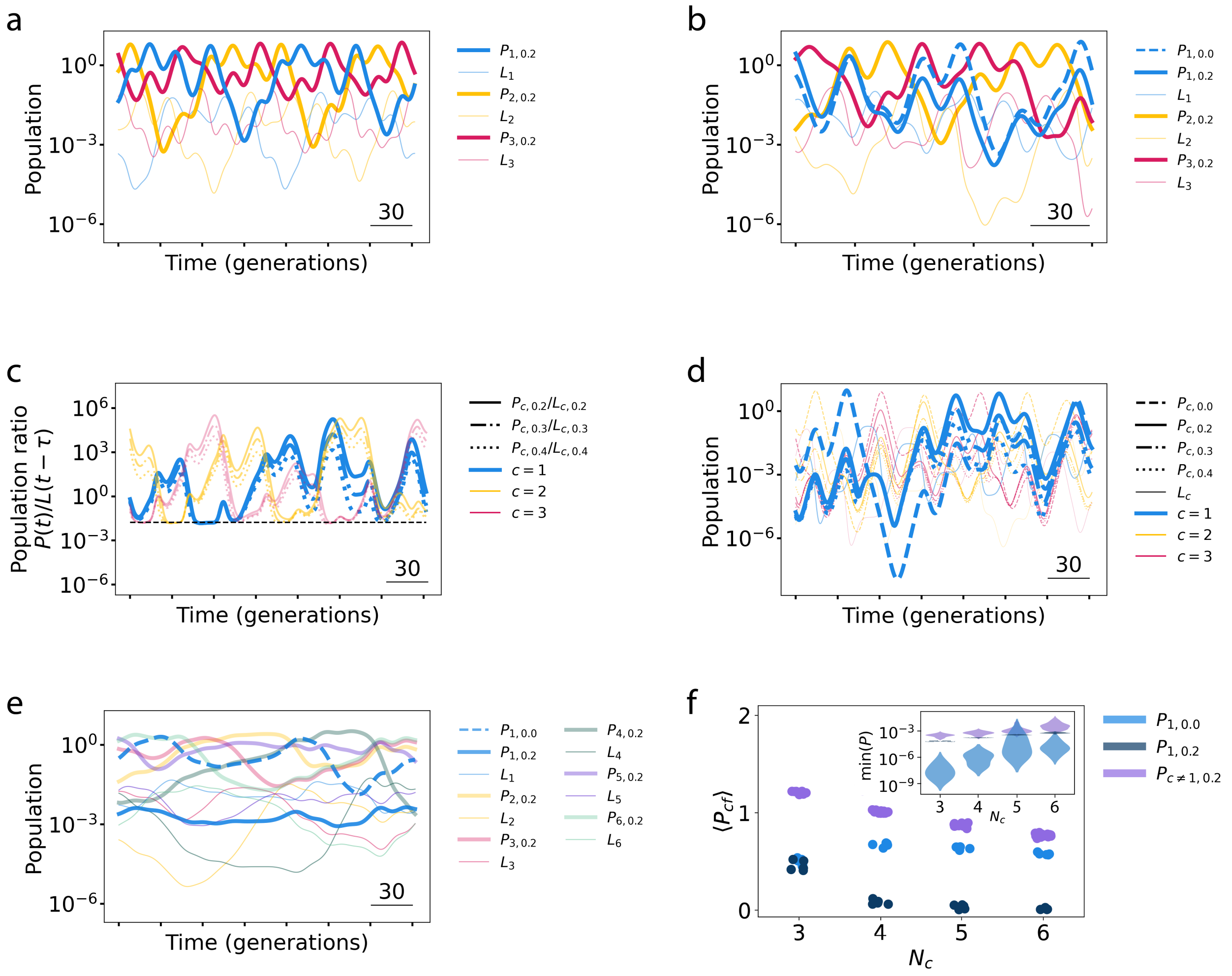

Figure S8: **Recapitulation of Fig. 2 incorporating a finite lysis time.** All panels as in Fig. 2, using equations (S5) in place of equations (1). $K = 0.2$ and $k = 1/N_c$ throughout; see Section S3. In panel c, phage population densities at time t are compared to lysogens at time $t - \tau$ where $\tau \approx 2.5$ generations is the time delay between phage infection and lytic burst; see Fig. S14 for ratio of $P(t)/L(t)$. $N_c = 2$ is not included in panel f as it leads to quasi-oscillatory solutions. Panel f shows summary statistics across 5 simulations, simulated for 5×10^3 generations each.

S1 Parameters

The values for the parameters used in the model are motivated by Cortes *et al.* (2019) [1]. Parameters setting the units for time and population density are the lysogen growth rate α and the infection rate constant k, which are both set to unity. The induction rate is small compared to the lysogen growth rate: $\gamma = 10^{-4}\alpha$. With this value of γ, induction of one lysogenic offspring will occur roughly 12 generations after lysogeny.

Our parameter choices differ from those of Cortes *et al.* in three main ways. First, while we consider finite nutrient conditions (and thus finite bacterial carrying capacities) in Section S2, we simplify the model in the main text to assume bacterial populations are exclusively limited by phage predation. Second, we assume a higher phage degradation rate than Cortes *et al.* by setting $\delta = \alpha$, accounting for both phage degradation and migration out of the local environment.

The third way our parameter choices differ from those of Cortes *et al.* regards the lysis time. Once a phage decides to undergo lysis, it creates many new phage virions. This process takes a significant amount of time, of the same order as a typical bacterial generation time. For example, phage λ has a period of ~ 50 minutes between infection and lysis of its *E. coli* host, roughly 2.5 times as long as the doubling time of *E. coli* in the same experiment (20 minutes) [2]. Below, we describe how a model with zero delay and a small burst size leads to the same overall phage growth rate as a model with a finite delay and larger burst size.

To model a finite lysis time explicitly, we could in principle modify equation (1) into a delay differential equation with a delay time τ:

$$\frac{dP_{c0}(t)}{dt} = k(b_\tau - 1)P_{c0}(t-\tau)\sum_{c' \neq c} L_{c'}(t-\tau) - \delta P_{c0}(t), \tag{S1}$$

where for simplicity, we have considered the obligate lytic phage ($f = 0$), and assumed it is the only strain in immunity class c. We have denoted the burst size in this model by b_τ to distinguish it from the burst size in the model with $\tau = 0$. Also for simplicity, we approximate the total lysogen population density susceptible to our strain of interest by a constant, L. We then have:

$$\frac{dP_{c0}(t)}{dt} = k(b_\tau - 1)LP_{c0}(t-\tau) - \delta P_{c0}(t). \tag{S2}$$

The solution to this equation approaches an exponential for large t. Keeping all other parameters equal (here we use $L = 0.2$), we find that the solution for finite τ is well-approximated by a model with $\tau = 0$ and a smaller burst size $b \equiv b_0$. The dependence of b on the time-delayed burst size b_τ and the length of the delay τ is shown in Fig. S9a. For realistic parameters, $\tau = 51$ min and $b_\tau = 170$ [2], we obtain an equivalent model with $\tau = 0$ and burst size $b = 13$ (rounded up from a best-fit value of 12.5). This simplified model with $\tau = 0$ gives a very good approximation to the results with a finite value of τ and a larger burst size, as shown in Fig. S9b. For the results of a model with finite lysis time and a corresponding large burst size, see Section S3.

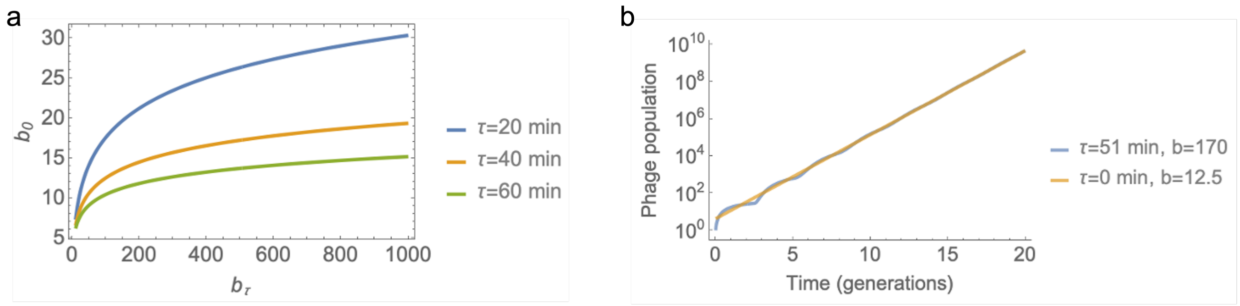

Figure S9: **A model with zero lysis time and a small burst size quantitatively approximates one with a finite lysis time and a larger burst size. a,** As a function of the true burst size b_τ for a given lysis time τ, the best-fit burst size for a model with zero lysis time, b_0, is shown. **b,** The solution to the delay-differential equation with $\tau = 51$ min and $b_\tau = 170$ is plotted alongsize the solution with $\tau = 0$ and $b_0 = 12.5$.

Parameter	Symbol	Default value	Unnormalized units
Infectivity	k	1	$(tc)^{-1}$
Lysogen growth rate	α	1	t^{-1}
Phage death rate	δ	1	t^{-1}
Induction rate	γ	10^{-4}	t^{-1}
Lysogeny fraction	f	0.2	None
Burst size	b	13	None

Table S1: **Parameters and their values (unless specified otherwise).** α and k set the units of time and population density; by setting these to unity, parameters are kept unitless. In the absence of such normalization, units would be given by the third column, where t represents units of time, and c units of concentration.

The full set of parameters used in our study is given in Table S1. In order to explore the robustness of our results to changes in these parameters, we performed one-dimensional parameter sweeps, exploring how coexistence and population density sizes changed in response to parameter variation. We used the $N_c = 6$ system, and kept all parameters but one as described in Table S1. One by one, we varied the following parameters: the lysogeny fraction f of all temperate phage; the lysogeny fraction f of temperate phage sharing an immunity class with an obligate lytic strain; the infectivity k, the induction rate γ, the burst size b, and the phage death rate δ. The results are shown in Fig. S10 and demonstrate robustness to parameter changes.

We additionally explored how the nature of coexistence – steady-state, oscillatory, or chaotic – changed as a result of parameter changes (Fig. S10a). We found robustness of the chaotic behavior observed to parameter changes, with the apparent exception of δ: varying δ by an order of magnitude in either direction yields oscillatory results for simulations lasting $> 10^4$ generations. To explore this behavior further, we measured the effect of more incremental changes in δ within this two-order-of-magnitude range, as well as the effect of changing the simulation time; results are shown in Fig. S11. For each δ and for a given simulation time of T generations, we examined the results in a window of $\min\{T, 420\}$ generations for evidence of oscillatory behavior, for 9 different initial conditions. Panels b-c of Fig. S11 show example chaotic trajectories for values of δ varying by nearly two orders of magnitude.

Initial conditions were chosen to be close to the predicted steady-state value while allowing for reasonable variation, as follows: Given the analytically predicted fixed point $(P^\star, L^\star)$ in the absence of obligate lytic phage, the initial conditions were given by multiplying $(3P^\star, L^\star)$ by a uniformly chosen random number between 0 and 1, and further multiplying P by a logarithmically chosen random number between 10^{-1} and 10^1. The 9 initial conditions used were kept consistent throughout Fig. S11. This procedure was used to generate initial conditions throughout the manuscript.

Finally, we explored how heterogeneity in the parameters can affect coexistence. Using the same $N_c = 6$ system, we varied the parameters b, k, f, and γ randomly – and independently for each phage/lysogen pair – from the general values in Table S1. We tested 10 random parameter sets at each one of 11 chosen fractional spreads ranging from 1% variation to 50%. For a given fractional spread σ, parameter values were chosen uniformly from within the range $1 \pm \sigma$ times their default value. Our results, shown in Fig. S12, show robust coexistence in these heterogeneous parameter systems. With 10% parameter variation, 9/10 simulations showed coexistence of all strains after ~14,000 generations. Even with 50% parameter variation, an average of 4 phage strains continue to coexist after ~14,000 generations.

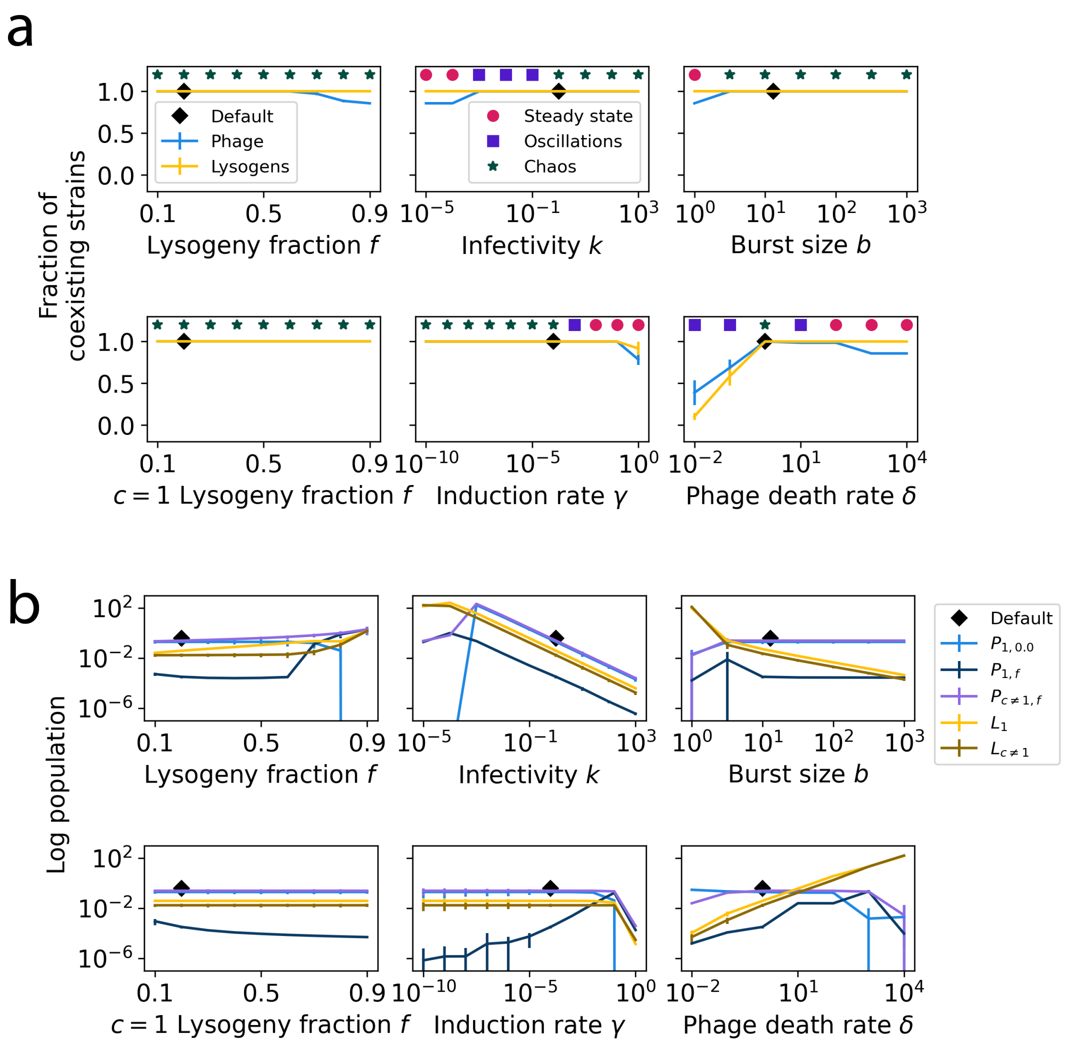

Figure S10: **One-dimensional parameter sweeps show robustness of model results to parameter changes.** We simulated the $N_c = 6$ system, with 5 immunity classes each consisting of a single temperate strain, along with a single class $c = 1$ with two strains (one obligate lytic, one temperate). Each parameter combination was simulated with 10 random initial conditions, for 10^4 timesteps (i.e. ∼14,500 generations) each. Panel **(a)** shows the fraction of strains coexisting at the end of each simulation, and the nature of the coexistence for each parameter tested (steady state: red circles; oscillatory: purple squares; chaotic: green stars); panel **(b)** shows the average population densities. Error bars show the standard error of the mean. The default parameter values used in the main text are indicated by black diamonds.

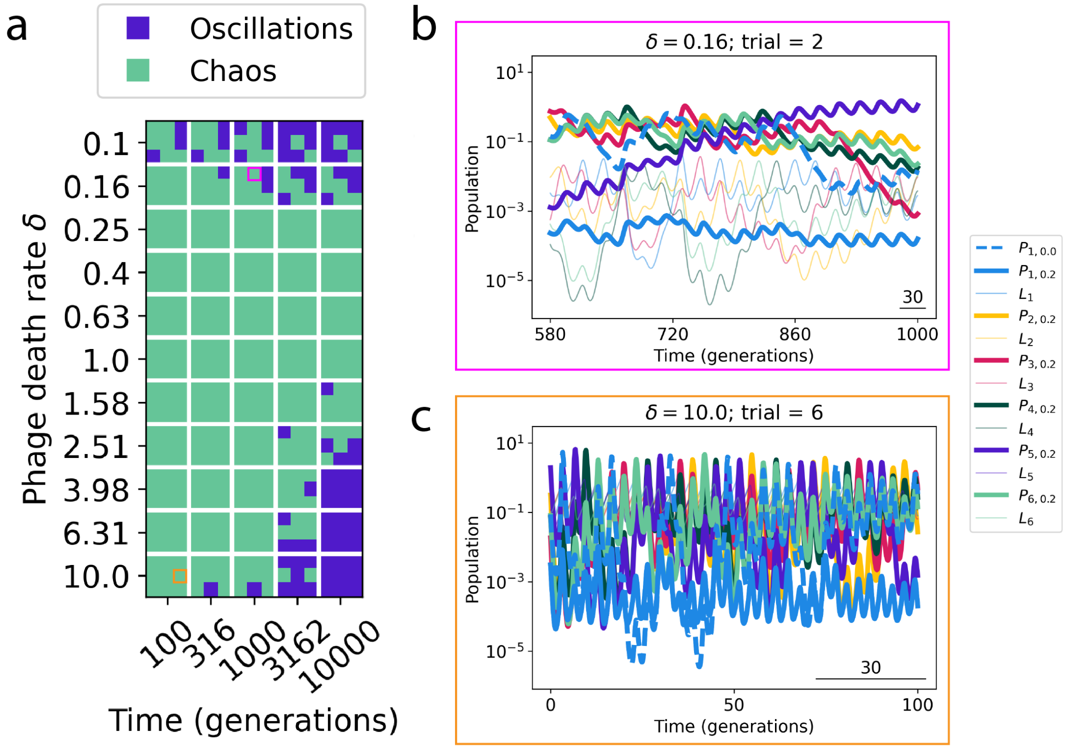

Figure S11: **Robustness of chaotic behavior observed to changes in phage death rate δ and total simulation time.** Simulations were performed for different values of δ as in Fig. S10. For each δ, simulations of 5 different lengths were performed, for 9 different initial conditions. (For each of $\delta = 0.1$ and 0.16, one initial condition led to a global extinction event; these two simulations were therefore rerun with different initial conditions.) Panel **a** shows all results for the dynamics pertaining at the end of the simulations, with each set of 9 replicates represented by a 3x3 grid. Light green squares represent chaotic trajectories; purple squares represent oscillatory trajectories. Panels **b-c** show two example trajectories, corresponding to the magenta and orange squares in panel a.

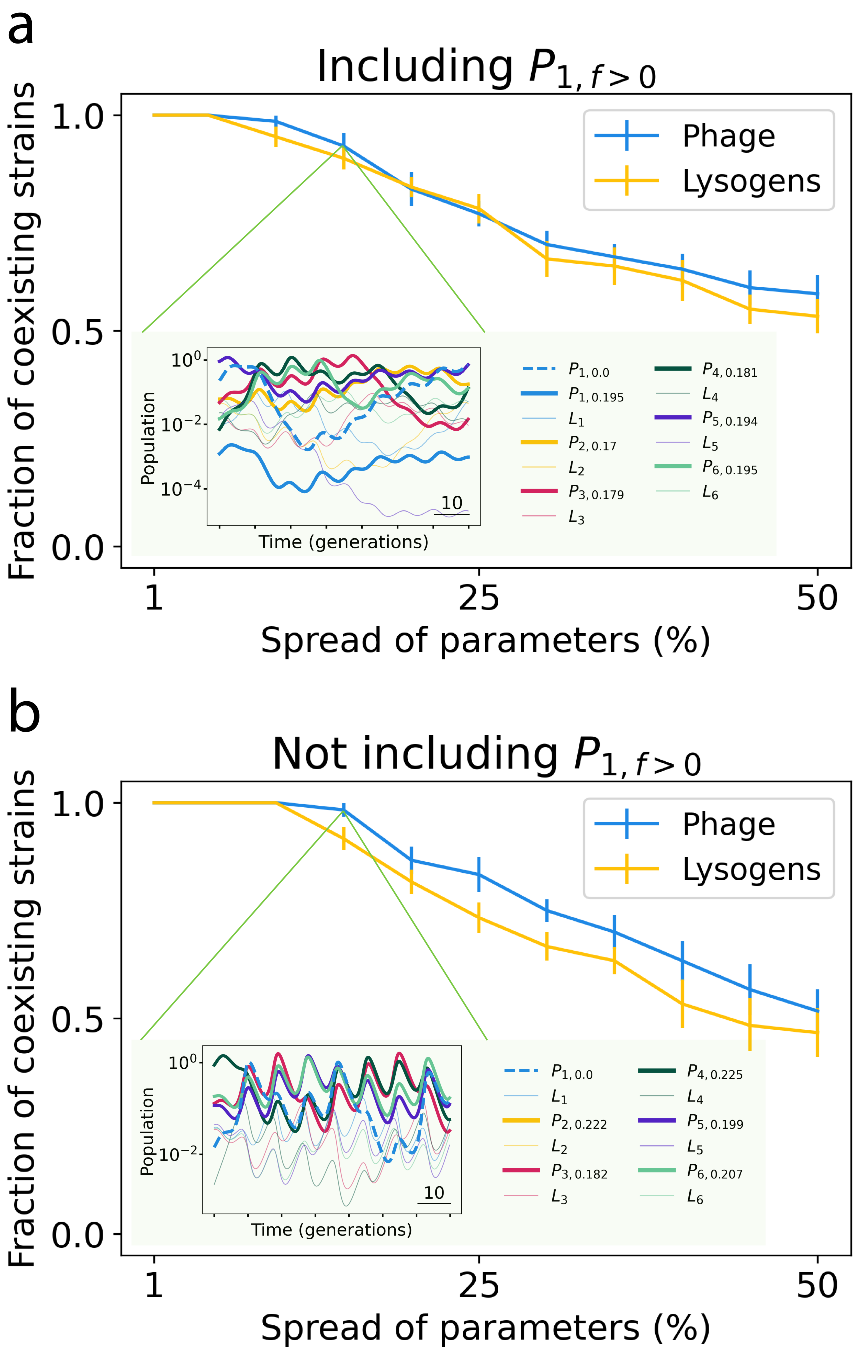

Figure S12: **Coexistence with random heterogeneous parameters.** We simulated the $N_c = 6$ system, with 5 classes each consisting of a single temperate strain, along with a single class $c = 1$ with an obligate lytic strain. **Panel a** shows results wherein class $c = 1$ includes both an obligate lytic and a temperate strain; **panel b** shows results wherein class $c = 1$ includes only an obligate lytic strain, with non-lysogenic bacteria L_1 having induction rate $\gamma = 0$ and being immune to infection by phage $P_{1,0}$. Parameters f, k, b, γ, and α were all varied randomly and uniformly from the values listed in Table S1 by between 1% and 50%. Parameters were varied heterogeneously and separately for each phage, lysogen, and phage-lysogen interaction. The fraction of strains coexisting after 10^4 timesteps (i.e. $\sim$14,500 generations) is shown. Example simulations with random parameter variations of 15% are shown. Error bars represent standard deviations across 10 trials.

S2 More comprehensive model

The model considered in the main text is a natural simplification of a more comprehensive model including both sensitive (i.e. non-lysogenic) bacteria and double lysogens. In this section, we discuss this more comprehensive approach. Main qualitative results are unchanged between the models (Fig. S6).

This model is summarized in Fig. S1, which pictorally depicts the following set of equations:

$$\begin{aligned}
\kappa =& 1 - \Big(S + \sum_c L_c + \frac{1}{2}\sum_{c,c'} D_{c,c'}\Big)/K, \\
\frac{dS}{dt} =& \left(\alpha_S \kappa - \delta_b\right) S - kS\sum_c P_c + r\sum_c L_c, \\
\frac{dL_{cf}}{dt} =& \left(\alpha\kappa - \delta_b\right) L_{cf} + kfSP_{cf} - (\gamma + r)L_{cf} - kL_{cf}\sum_{c'\neq c} P_{c'} + r\sum_{c'} D_{cf,c'}, \\
\frac{dD_{cf,c'f'}}{dt} =& \left(\alpha_D\kappa - \delta_b\right) D_{cf,c'f'} + kfL_{c'f'}P_{cf} + kf'L_{cf}P_{c'f'} \\
& - D_{cf,c'f'}\Big[2(\gamma + r) + k\sum_{c''\neq c,c'}\sum_{f''}(1 - f'')P_{c''f''}\Big], \\
\frac{dP_{cf}}{dt} =& b\gamma\Big(L_{cf} + \sum_{c'} D_{cf,c'}\Big) + \\
& P_{cf}\Bigg(- k\Big[S + \sum_{c'}(L_{c'} + \frac{1}{2}\sum_{c''} D_{c',c''})\Big] - \delta + kb\left(1 - f\right)\Big[S + \sum_{c'\neq c}(L_{c'} + \frac{1}{2}\sum_{c''\neq c} D_{c',c''})\Big]\Bigg),
\end{aligned} \tag{S3}$$

where we have defined a reversion rate r for a lysogen to lose a prophage (e.g. via mutation), a carrying capacity K for bacteria (which implies an associated population-dependent growth-rate modification factor κ), a bacterial death rate δ_b, and different growth rates α_S, α, and α_D for the sensitive bacteria S, single lysogens L, and double lysogens D, respectively. The double lysogens are defined such that $D_{cf,c'f'}$ is the population density of double lysogens created by the infection of a sensitive bacterium by both P_{cf} and $P_{c'f'}$. Infection can occur in any order, such that $D_{c,c'} = D_{c',c}$ (and $D_{c,c} = 0$). As previously, we have omitted the subscript f to denote summing over the different strains of a given immunity class.

For clarity, we will now describe the origin of each term in the equation for dD/dt. The first term represents the growth and spontaneous death of double lysogens. Bacterial growth is logistic: the growth rate is reduced as bacteria become more populous and is zero at the carrying capacity K. The second and third terms represent the creation of double lysogens *via* the lysogeny of either of the two single lysogens. The fourth term represents the removal of double lysogens from the population through various means, and is itself split into separate terms: removal due to induction or reversion of either of the two lysogens; and removal due to infection and subsequent lysis by phage of a separate immunity class.

We assume that lysogens incur a small growth rate penalty, setting $\alpha_S = 1.05$ and $\alpha_D = 0.95$ (with $\alpha = 1$) [1]. We set the reversion rate r equal to the induction rate γ. Since reasonable values of the bacterial death rate δ_b are small enough that this term is negligible compared to bacterial death due to phage predation, we set $\delta_b = 0$. The results of simulations of these full equations for different values of N_c, with $K = 100$ (simulating non-limiting nutrient conditions), are shown in Fig. S6 (right panel). In these simulations, we set one immunity class to have both obligate lytic and temperate strains, and the other $N_c - 1$ immunity classes to only have a single temperate strain.

We note that this more comprehensive model lacks the perfect memory of initial lysogen populations implicit in the simplified model (equations (1)). This model yields the same qualitative results as our simplified main-text model (Fig. S7).

To recover the main-text equations from equations (S3), we make three approximations. The first of these is assuming that phage attempts to lysogenize a single lysogen result in the death of the phage rather than in the creation of a double lysogen (just as equations (S3) make an analogous approximation that neglects triple lysogens). The outcome of this approximation is that $D = 0$ for all strains, leading to the following set of equations (depicted in Fig. S13):

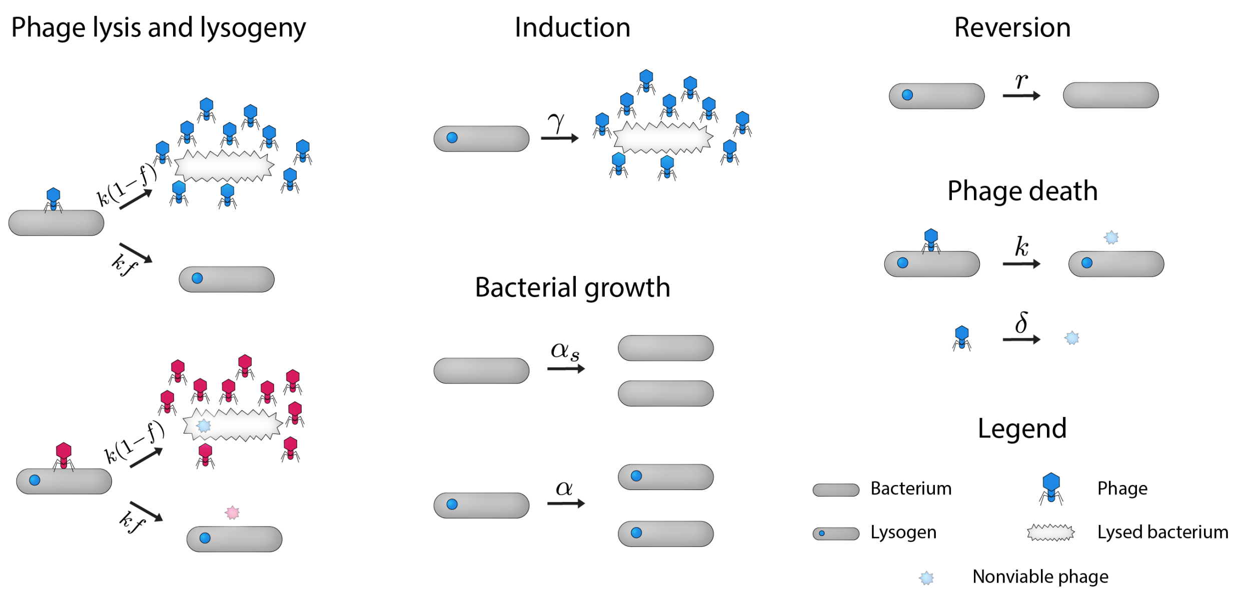

Figure S13: **Overview of model including sensitive bacteria.** A pictoral representation of the model described by equations (S4). Phage of different immunity classes are represented by different colors.

$$
\begin{aligned}
\kappa =& 1 - \Big(S + \sum_c L_c\Big)/K, \\
\frac{dS}{dt} =& \alpha_S \kappa S - kS \sum_c P_c + r \sum_c L_c, \\
\frac{dL_{cf}}{dt} =& \alpha \kappa L_{cf} + kfSP_{cf} - (\gamma + r)L_{cf} - kL_{cf} \sum_{c' \neq c} P_{c'}, \\
\frac{dP_{cf}}{dt} =& b\gamma L_{cf} + P_{cf}\left(-k\Big[S + \sum_{c'} L_{c'}\Big] - \delta + kb\,(1-f)\,\Big[S + \sum_{c' \neq c} L_{c'}\Big]\right).
\end{aligned} \tag{S4}
$$

Results of simulations of these equations can be seen in Fig. S6 (middle panel). This simplification yields the same qualitative picture as the full model, with one major exception: in the full model (including double lysogens), $N_c = 2$ behaves like $N_c = 1$ in the single-lysogen model. In both cases, a single bacterial strain immune to all phage takes over the bacterial population, and leads to the extinction of obligate lytic phage strains, while temperate strains survive as a result of induction. Furthermore, $N_c = 3$ in the double-lysogen model behaves qualitatively like $N_c = 2$ in the single-lysogen model: both sometimes show oscillatory or quasi-oscillatory behavior. Finally, $N_c = 4$ in the double-lysogen model is qualitatively akin to $N_c = 3$ in the single-lysogen model: both display chaotic behavior. Thus, the model system displays chaotic behavior when there are at least two more immunity classes than the maximum allowed number of cohabiting prophage within lysogens. More generally, we find that the full model with N_c immunity classes behaves similarly to the model disallowing double lysogens with $N_c - 1$ classes.

The other two approximations made to the full model to recover the main-text equations are to: 1) assume infinite K (such that $\kappa = 1$), and 2) to set $S = 0$ (assuming zero reversion). Both are motivated by the results shown in Fig. S6, which demonstrate that: 1) predation by phage limits bacterial populations, and 2) the population of sensitive bacteria is negligible, especially for $N_c > 2$. As shown in Fig. S6 (left panel), these simplifications do not affect the qualitative behavior of the system.

S3 Finite lysis time model

In the main text, we considered a model wherein lysis is immediate. However, in nature, lysis typically occurs a time τ following infection, where $\tau \approx 2.5$ bacterial generations [2]. We therefore sought to consider a model implementing this time delay, and including a burst size of $b = 170$ (see Fig. S9). Following Ref. [3], we implemented a time delay by 10 intermediate infected states; this intermediate state model leads phage to produce a burst typically a time τ after infection (Fig. S14a). Defining $I_{cf,c'f',l}$ as the lth intermediate state resulting from lytic infection of lysogen L_{cf} by phage $P_{c'f'}$, the resulting equations for this time delay model are:

$$\begin{aligned}
\kappa =& 1 - \Big(\sum_{c,f} L_{cf} + \sum_{c,f,c',f',l} I_{cf,c'f',l}\Big)/K, \\
\frac{dP_{cf}}{dt} =& \frac{10}{\tau} b \sum_{c'f'} I_{c'f',cf,10} - P_{cf}\left[\delta + k \sum_{c'f'}\left(L_{c'f'} + \sum_{c''f''l} I_{c'f',c''f'',l}\right)\right], \\
\frac{dL_{cf}}{dt} =& L_{cf}\left(\alpha\kappa - \gamma - k \sum_{c'\neq c,f'} (1-f')P_{c'f'}\right) + k \sum_{c',f'} I_{cf,c'f',1}\Theta(f'>0)\sum_{f''} P_{c'f''}, \\
\frac{dI_{cf,c'f',1}}{dt} =& k(1-f')P_{c'f'}L_{cf}(1-\delta_{c,c'}) + \gamma L_{cf}\delta_{c,c'}\delta_{f,f'} - I_{cf,c'f',1}\left(\frac{10}{\tau} + k\Theta(f'>0)\sum_{f''} P_{c'f''}\right), \\
\frac{dI_{cf,c'f',l>1}}{dt} =& \frac{10}{\tau}\left(I_{cf,c'f',l-1} - I_{cf,c'f',l}\right),
\end{aligned} \tag{S5}$$

where we have defined a carrying capacity K for bacteria (which implies an associated population-dependent growth rate modifier κ), the Kronecker delta function $\delta_{c,c'}$ which is unity if $c = c'$ and zero otherwise, and further define the Heaviside theta function $\Theta(x > 0)$ to be unity if $x > 0$ and zero otherwise. The last terms in dL_{cf}/dt and $dI_{cf,c'f',1}/dt$ express a multiplicity-of-infection feature of this model. Since phage co-infection has been shown to favor lysogeny over lysis [4, 5], in our model, when a phage infects the first intermediate state of a lytic infection caused by a temperate phage of that same immunity class, that phage infection changes from the lytic pathway to the lysogenic. As in the main text model, attempts to form a double lysogen lead to death of the infecting phage; therefore, multiplicity of infection leads the infected bacterium to revert to its pre-infection lysogenic state. A phage infecting any later intermediate state of a lytic infection has no effect on the bacterium, but results in loss of the phage.

A finite carrying capacity K is an essential feature of this time-delay model. Without a finite carrying capacity, we find that the continued growth of phage for several generations after bacteria populations start decaying typically leads to bacterial population collapse. A finite carrying capacity ensures that the amplitudes of the chaotic dynamics do not grow too large.

A recapitulation of Fig. 2 using these time-delay equations, with $b = 170$ (see Section S1) and $K = 0.2$, is shown in Fig. S8. We find that unlike in the main-text model, total lysogen population changes significantly as a function of N_c. In order to maintain that each phage has an equal probability per unit time of infecting a cell at all N_c's, we scale infectivity k with the number of phage immunity classes N_c as $k = 1/N_c$; no such rescaling was necessary for our main-text model (Fig. S14c-d).

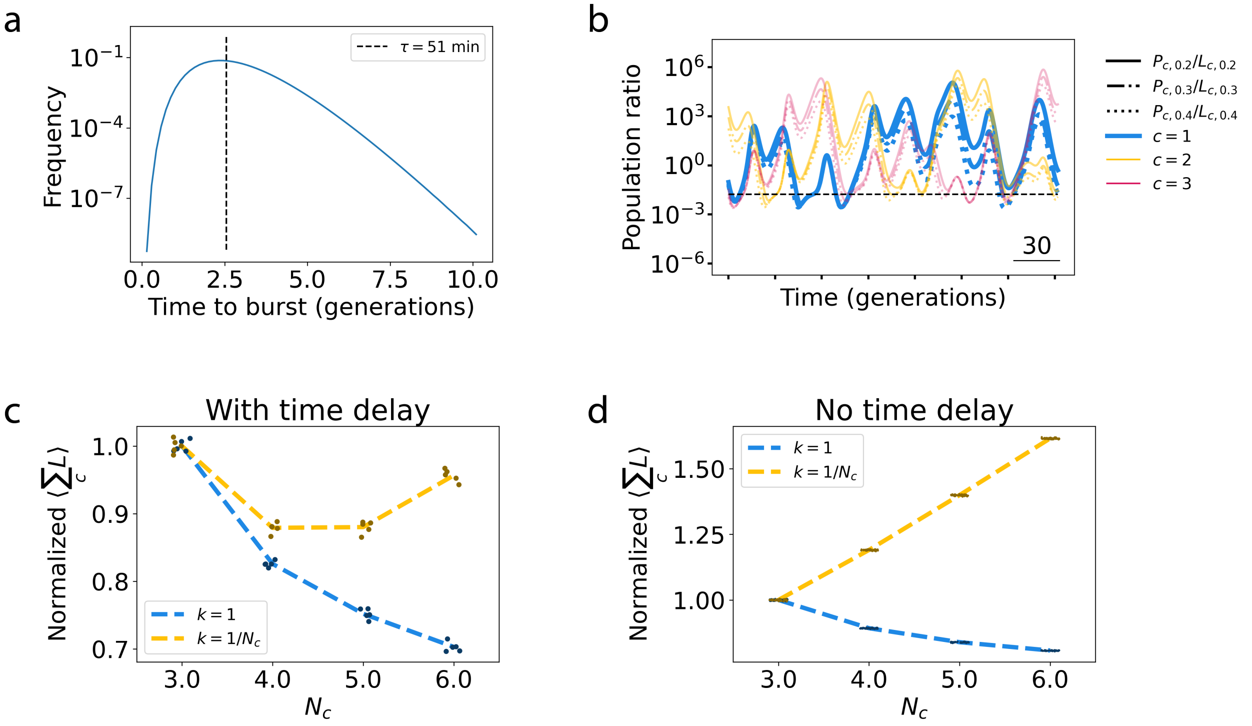

Figure S14: **Details of finite lysis time model. a,** Histogram of the time after infection leading to a burst of $b = 170$ phage. Vertical dashed line is at $\tau \approx 2.5$ bacterial generations. **b,** Ratio of population densities of phage and their corresponding lysogens, as in Fig. S8c, but where phage and lysogen population densities are compared at the same time, $P(t)/L(t)$ (rather than $P(t)/L(t-\tau)$ which is shown in Fig. S8c). **c,d** The average total lysogen population density is displayed as a function of N_c when infectivity k is either constant or scaled as $1/N_c$. For ease of comparison, lysogen population density is normalized by its average value at $N_c = 3$. Panel c shows results for the finite lysis time model (equations (S5)); panel d shows results for the main-text model (equations (1)).

S4 Chaotic behavior

Chaotic population trajectories are the predominant behavior found in the simplified model for $N_c \geq 2$, though not the only possibility: both a static fixed point and periodic oscillations sometimes occur. However, the latter have very small basins of attraction. We did not observe convergence to a fixed point in any of our simulations, but the fixed point can be solved for analytically. While simulations starting at the fixed point remain there, those that start even 1-5% away from it display chaotic behavior.

Some trajectories with $N_c = 2$ enter an oscillatory regime (as discussed above). We have also found some trajectories with $N_c = 3$, with a single temperate strain of $f = 0.2$ in each immunity class, that converge to an oscillatory regime after $10^4 - 10^5$ generations. In this case, a sudden variation of 50% in the population densities returns the system to the chaotic regime. We have never observed periodic oscillations upon introducing an obligate lytic strain to these $N_c = 3$ simulations (Fig. S15a) nor do any of the trajectories in Fig. 2f show evidence of periodic oscillations. Similarly, we never observed periodic oscillations upon introducing a fourth identical phage immunity class (Fig. S15b), nor with $N_c = 3$ immunity classes where each strain has a different lysogeny fraction (Fig. S15c).

In Fig. S16, we show various return maps, plotting how each extremum in the total phage or total lysogen population density of an $N_c = 2$ system compares to the next extremum of that population density (using the Lorenz system as a guide). The results display a fractal dimension of ~ 1.3 as calculated using a box-counting algorithm [6], consistent with chaotic dynamics.

Given that natural variations in environmental conditions would frequently induce variability into the system, and given the natural heterogeneity among different phage populations, we do not expect periodic oscillations to be biologically relevant for $N_c > 2$.

S5 Brief derivation of Eq. (3)

Here, we derive P^{ss}_{cf}, the steady-state value of P_{cf} subject to the dynamics of Eq. (1). Specifically, we assume the lysogen dynamics have reached steady state at a non-zero lysogen population L_{cf}. In this case, we have

$$\alpha - \gamma = k \sum_{c' \neq c} \sum_{f'} (1 - f') \, P^{\text{ss}}_{c'f'}. \tag{S6}$$

We consider the symmetric case in which all lysogeny fractions f' are equal and given by $f' = f$. Assuming parameter symmetries, the steady-state values of the phage population densities will be equal for different immunity classes, such that $\sum_{c' \neq c} \sum_{f'} (1 - f') \, P^{\text{ss}}_{c'f'} = (1 - f) \, (N_c - 1) \, P^{\text{ss}}_{cf}$, where N_c is the number of phage immunity classes. We thus arrive at Eq. (3),

$$P^{\text{ss}}_{cf} = \frac{\alpha - \gamma}{k(N_c - 1)(1 - f)}. \tag{S7}$$

References

[1] Michael G. Cortes, Jonathan Krog, and Gábor Balázsi. Optimality of the spontaneous prophage induction rate. Journal of Theoretical Biology, 483, 2019.

[2] Ing Nang Wang. Lysis timing and bacteriophage fitness. Genetics, 172(1):17–26, 2006.

[3] Rasmus Skytte Eriksen, Namiko Mitarai, and Kim Sneppen. Sustainability of spatially distributed bacteria-phage systems. Scientific reports, 10(1):3154, 2020.

[4] Myron Levine. Mutations in the temperate phage P22 and lysogeny in Salmonella. Virology, 3(1):22–41, 1957.

[5] Tianyou Yao, Seth Coleman, Thu Vu Phuc Nguyen, Ido Golding, and Oleg A. Igoshin. Bacteriophage self-counting in the presence of viral replication. Proceedings of the National Academy of Sciences of the United States of America, 118(51), 2021.

[6] Francesco Turci. Box Counting in Numpy. https://francescoturci.net/2016/03/31/box-counting-in-numpy/, 2016.

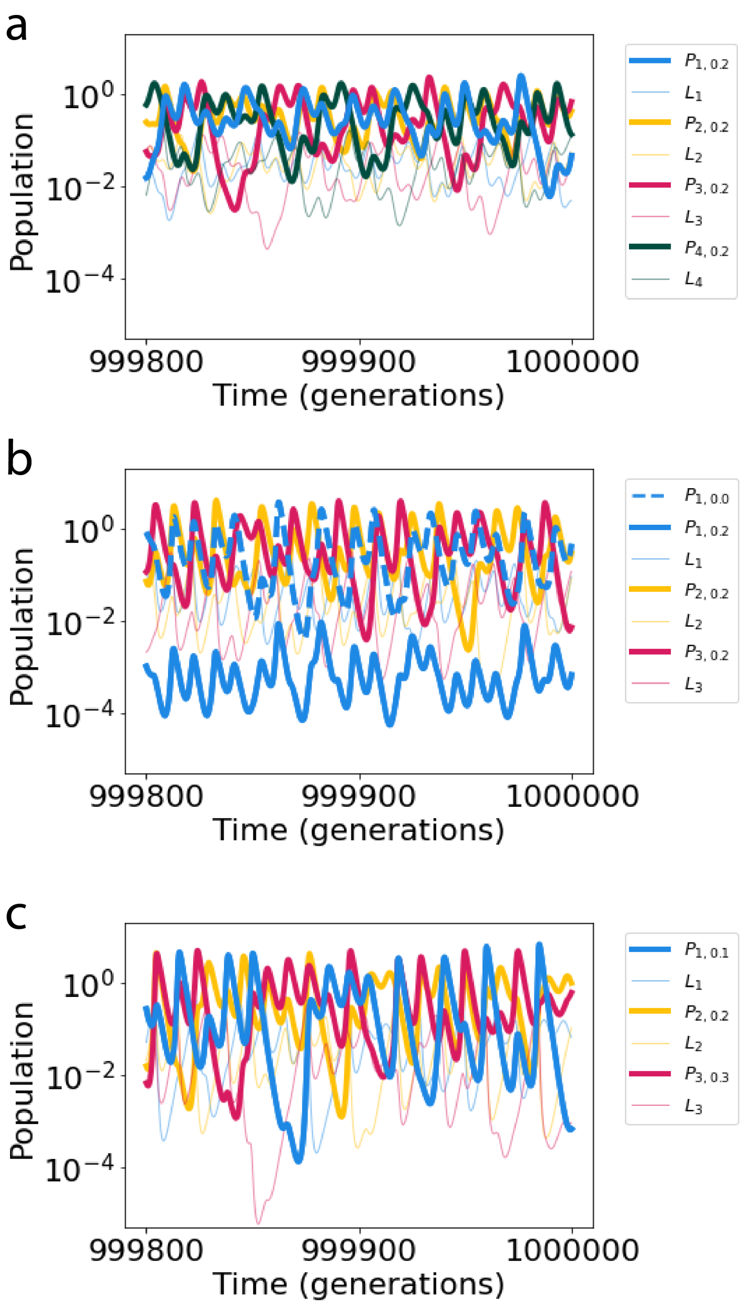

Figure S15: **Chaotic trajectories are typical. a,** Simulation with $N_c = 4$ phage immunity classes, each with a single temperate strain with lysogeny fraction $f = 0.2$. **b,** Simulation with $N_c = 3$ phage immunity classes, one of which has two strains—one obligate lytic and one temperate ($f = 0.2$)— and two of which have a single temperate strain ($f = 0.2$). **c,** Simulation with $N_c = 3$ phage immunity classes, each with a single temperate strain of different lysogeny fractions.

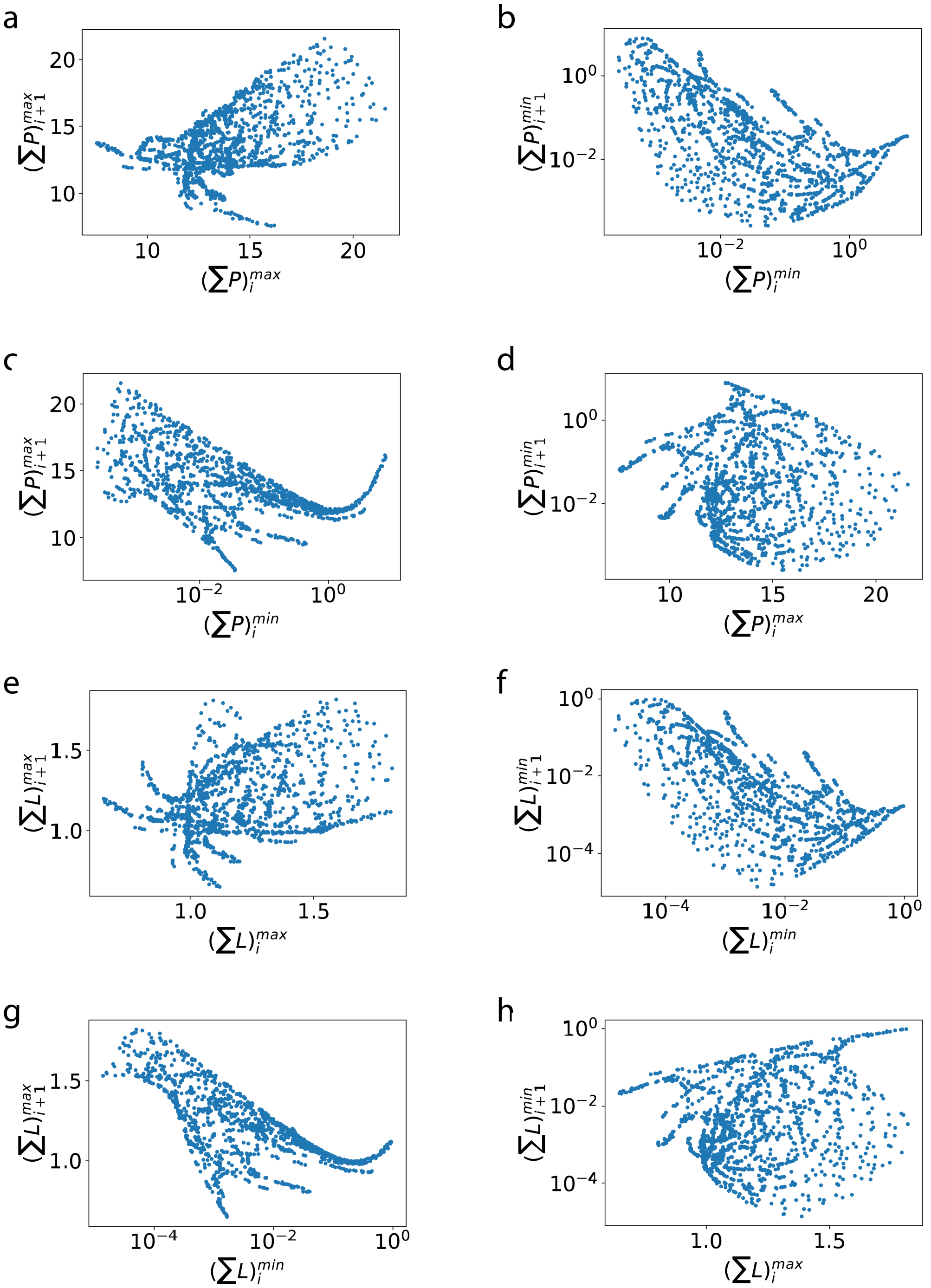

Figure S16: **Return maps for an $N_c = 2$ system, using equations** (1). **a,** Comparison of each maximum of total phage population density to the next maximum. **b,** Comparison of each minimum of total phage population density to the next minimum. **c,** Comparison of each minimum of total phage population density to the next maximum. **d,** Comparison of each maximum of total phage population density to the next minimum. **e,** Comparison of each maximum of total lysogen population density to the next maximum. **f,** Comparison of each minimum of total lysogen population density to the next minimum. **g,** Comparison of each minimum of total lysogen population density to the next maximum. **h,** Comparison of each maximum of total lysogen population density to the next minimum.
